# Sensitivity analysis in multiple imputation in effectiveness studies of psychotherapy

**DOI:** 10.3389/fpsyg.2015.01042

**Published:** 2015-07-27

**Authors:** Aureliano Crameri, Agnes von Wyl, Margit Koemeda, Peter Schulthess, Volker Tschuschke

**Affiliations:** ^1^School of Applied Psychology, Zurich University of Applied SciencesZurich, Switzerland; ^2^Swiss Charta for PsychotherapyStäfa, Switzerland; ^3^Division of Medical Psychology, University Hospital of CologneCologne, Germany; ^4^Faculty of Psychotherapy Sciences, Sigmund Freud UniversityBerlin, Germany

**Keywords:** multiple imputation, sensitivity analysis, outpatient psychotherapy, quality assurance, therapeutic alliance, routine outcome monitoring, OQ-45, HAQ

## Abstract

The importance of preventing and treating incomplete data in effectiveness studies is nowadays emphasized. However, most of the publications focus on randomized clinical trials (RCT). One flexible technique for statistical inference with missing data is multiple imputation (MI). Since methods such as MI rely on the assumption of missing data being at random (MAR), a sensitivity analysis for testing the robustness against departures from this assumption is required. In this paper we present a sensitivity analysis technique based on posterior predictive checking, which takes into consideration the concept of clinical significance used in the evaluation of intra-individual changes. We demonstrate the possibilities this technique can offer with the example of irregular longitudinal data collected with the Outcome Questionnaire-45 (OQ-45) and the Helping Alliance Questionnaire (HAQ) in a sample of 260 outpatients. The sensitivity analysis can be used to (1) quantify the degree of bias introduced by missing not at random data (MNAR) in a worst reasonable case scenario, (2) compare the performance of different analysis methods for dealing with missing data, or (3) detect the influence of possible violations to the model assumptions (e.g., lack of normality). Moreover, our analysis showed that ratings from the patient's and therapist's version of the HAQ could significantly improve the predictive value of the routine outcome monitoring based on the OQ-45. Since analysis dropouts always occur, repeated measurements with the OQ-45 and the HAQ analyzed with MI are useful to improve the accuracy of outcome estimates in quality assurance assessments and non-randomized effectiveness studies in the field of outpatient psychotherapy.

## Introduction

Missing data that occur if patients drop out, either from the treatment or from the whole study, are a serious source of bias in the evaluation of treatment effectiveness. There are numerous works demonstrating the implementation of multiple imputation (MI) in the analysis of longitudinal data with missing values (Enders, 2011; Graham, 2012; Van Buuren, 2012). However, some questions remain open, when this technique is applied to data collected in a practice setting, as in the case of quality assurance programs or effectiveness studies with a focus on the estimation of intra-individual changes (Beutler, 2001). Effectiveness studies of psychotherapy are observational (Rosenbaum, 2010) and focus on external validity: data collection is carried out in a practice setting, patients are not randomized between different treatment conditions, and a rigorous treatment manual is not imposed to the therapists who can freely adapt their interventions to the needs of their patients (Seligman, 1995; Westbrook and Kirk, 2005). In contrast, efficacy studies, also called randomized clinical trials (RCT), emphasize the internal validity by means of highly controlled and ideal settings: patients are randomized and therapists have to adhere to a defined treatment manual. The topic of inference with missing data have been extensively researched in RCT, primarily conceived for testing pharmaceutical drugs (Little et al., 2012; Mallinckrodt, 2013). However, this knowledge can only be partially transferred to effectiveness studies of psychotherapy due of two peculiarities: the flexible length of the treatment and the lack of a control group.

The most commonly used approaches for MI of longitudinal data in clinical trials or epidemiological studies assume a common number of measurement occasions. Each time point is recorded as a separate variable in a *wide format* and imputed in the same way as cross-sectional data (Grittner et al., 2011; Kleinke et al., 2011; Ferro, 2014). This can easily be accomplished using routines offered by statistical software packages. However, the appropriateness of this approach is not obvious in case subjects are measured on a varying number of occasions. This is usually the case for outpatients receiving psychotherapy with a variable number of sessions over a variable time frame in a practice setting. For a patient, formally terminating therapy at session *t_i_*, non-existent missing values are coded for all variables representing sessions *t*_*j* > *i*_ in a wide format data set. Irregular longitudinal data can however, when they are arranged in a long format, be analyzed with random effects models. Van Buuren (2012) demonstrates how to generate multiple imputations from a linear mixed model based on a B-spline function using the example of body weight data collected on a variable number of occasions during the age between 0 and 29 years. The approach allows the analysis of irregular longitudinal data provided only that they cover a fixed overall time interval. Kurland et al. (2009) compared different mixed models applied to cohort data from aging research, in which losses due to death were common (this pattern of incompleteness can be considered analogous to that in effectiveness studies of psychotherapy). They pointed out that basically random effects models implicitly impute data beyond death and therefore need to be adapted in order to prevent this kind of estimation. The repercussion of imputing data after decease was investigated by Ning et al. (2013) who imputed all time waves for all subjects, including those deceased between waves, and then, before the analysis, discarding imputed values for the deceased after their death. They found that imputing values of subjects after their death influenced the imputations of survivors who had missing values, leading to inconsistent results. Altogether these findings lead to the conclusion that, standard imputation procedures need to be customized in order to treat longitudinal data measured on variable number of occasions and covering a variable period of time.

A further question relates to sensitivity to assumptions about the missing data mechanism. MI assumes that data are missing at random (MAR) (Little and Rubin, 2002), a condition that cannot be taken for granted. This condition is met if the probability of a value being missing depends on observed data, but given these, not on unobserved data. If, even after conditioning on observed data, the missingness of a variable still depends on the missing values of the variable itself, then these are classified as missing not at random (MNAR). The decision as to which of the two, MAR or MNAR, is valid in a specific case cannot be unequivocally determined through the analysis of available data, hence is based on the researcher's assumptions. According to the recommendations of the National Research Council (National Research Council, 2010), examining sensitivity of the results to these assumptions should be part of the primary statistical analysis in a clinical trial. Basically this sensitivity analysis consists in testing the robustness of results under a reasonable worst case scenario. Approaches developed for clinical trials use estimates derived from some reference group(s) (e.g., placebo condition) to impute the missing outcome data of dropouts in the treatment condition (Little and Yau, 1996; Carpenter et al., 2013; Ayele et al., 2014). However, to date there is still lack of guidelines on testing sensitivity to MNAR data in effectiveness studies without a control group.

In a longitudinal design the propensity to drop out is assumed to be related to the missing outcome values and therefore, the MAR assumption is justified if this relationship can be explained by data observed on preceding measurement occasions. However, the longer the intervals between these occasions, the less plausible is the assumption of MAR. Although MI is a procedure for MAR, it may, following Schafer (1997), perform well in cases when MNAR mechanisms are suspected, provided the richness of the available multivariate data and the complexity of the imputation model allow a good prediction of the variable affected by missing values. Concerning the effectiveness assessment, this means identifying strong predictors of the outcome itself and of the propensity to drop out. A self-report questionnaire used for predictive purposes in quality assurance of outpatient psychotherapy is the Outcome Questionnaire OQ-45 (Lambert et al., 2004). It is designed to be repeatedly administered during the course of treatment and has been suggested as an instrument for delivering to the therapist progress feedback, which can help in reducing treatment failures (Lambert, 2010). Questionnaires measuring the therapeutic alliance are another class of measures with a predictive value. One of these is the Helping Alliance Questionnaire (HAQ) (Luborsky, 1984). Findings from the meta-analytic review reported by Sharf et al. (2010) confirm that clients with weaker therapeutic alliance are at risk of dropping out of psychotherapy. From their meta-analytic review Martin et al. (2000) report consistent findings that the therapeutic relationship is positively associated, if moderately, with outcome in psychotherapy. On the other hand, Barber et al. (2010) cite several studies in which the temporal sequence between alliance development and symptom reduction was properly investigated and that failed to prove the predictive value of the alliance. For example, Strunk et al. (2010) found that the quality of the alliance was positively associated with the preceding symptomatic improvement, but that the alliance itself did not predict subsequent session-to-session symptom change. Given these findings, the question arises as to whether collecting additional data concerning the alliance quality can improve predictions based on routine outcome measures such as the OQ-45.

In the current paper we present a procedure to check the robustness of multiple imputations applied to outcome data from an effectiveness study. We will demonstrate the application on longitudinal data collected with the OQ-45 and the HAQ in routine clinical settings. Our analyses on this topic encompassed three objectives:

1. *Elaborating a way to impute missing values in longitudinal data measured on a varying number of occasions and covering varying time intervals*.
2. *Elaborating a procedure for sensitivity analysis in the evaluation of intra-individual changes*.
3. *Assessing the predictive value of the OQ-45 and the HAQ, as well as testing the performance of MI when applied to data collected by both questionnaires in a routine clinical setting*.

## Methods

### Sample and measures

The data were collected as part of a project promoted by the Swiss Charta for Psychotherapy, with the aim of investigating various process-outcome aspects of outpatient treatments (Tschuschke et al., 2015). In this non-randomized field study, therapies were carried out with different experiential and psychodynamic therapy methods. The research design was approved by the ethical committee of each Swiss canton, in which the study was carried out.

For the present analysis data from the first two assessments and from the process measurements were used. Both assessments, one at the beginning (pre) and one after the end of the therapy (post), included, among other things, structured diagnostic interviews based on DSM-IV (Wittchen et al., 1997) criteria and the completion of self-report questionnaires. One of these is the OQ-45, which measures three domains: symptom distress, interpersonal relations, and social role. The most sensitive scale of this instrument is OQ total, which is the sum score of all 45 items which measures the overall level of dysfunctionality. Based on our data, this scale had an internal consistency of Cronbach's α = 0.95. The OQ-45 together with the HAQ scale was filled in during the therapy after every 5th session (process measurements). The latter scale was applied in its patient version (HAQ-P, α = 0.88) as well as in its therapist version (HAQ-T, α = 0.89). In the present data analysis we additionally applied the factorial solution of De Weert-Van Oene et al. (1999) which divides the 11 items into two subscales: Cooperation (patient's version HAQ-P-C α = 0.90, therapist's version HAQ-T-C α = 0.87) and Helpfulness (patient's version HAQ-P-H α = 0.79, therapist's version HAQ-T-H α = 0.80).

For the present analysis data were used from therapies either terminated by mutual consent, or discontinued as a result of the patient's decision. The former are referred below as completers, the latter as dropouts. The percentages in Table 1 indicate a clear relationship between the type of termination and the outcome missingness. The imputation procedure used made possible the imputation of missing post-values from therapies with at least two process measurements. Hence, in the end, 91% of the sample could be included in the outcome analysis (*n* = 260). Participants were predominately female (67%). Among them, 10% had a low, 35% a middle, and 55% a high level of education. The most frequent Axis I diagnoses were affective (38%), anxiety (24%), and adjustment disorders (17%). A lifetime history of psychiatric disorders was present in 60% of the subjects. Two thirds of the sample had one or more current comorbidity disorders. The proportion of patients with Axis II disorders were 40%; of personality disorders (PD) diagnosed more of the half belonged to Cluster C. The mean duration of the treatments was 39 sessions (SD = 31). Therapies were monitored with measurements after each 5th session. At the beginning of the therapy the frequency was usually 1 treatment session per week and was lowered during treatment progress. The mean interval between the last three measurements was 14 and 17 weeks, respectively. Treatments were on average monitored by eight process measurements (min = 2, max = 32).

**Table 1:** **Type of termination and amount of missing outcome data in the original sample (*N* = 286)**.

	**Participation in the post-assessment**			**Total**
	**Yes**	**No**		
		**Imputable outcome data**	**Not imputable outcome data**	
Termination by mutual consent (completers)	181 (85%)	27 (13%)	4 (2%)	212 (100%)
Discontinuation decided by the patient (dropouts)	29 (39%)	23 (31%)	22 (30%)	74 (100%)
Total	210 (74%)	50 (17%)	26 (9%)	286 (100%)

*Missing outcome data is imputable if at least two process measurements are available*.

### Imputation procedure

Table 2 exhibits a summary of the proportion of missing values in the variables involved in the analysis. Since incompleteness is not only present in the target variable, i.e., the treatment outcome, but also in the predictors, a multivariate imputation approach is needed. The most commonly applied algorithms to generate multivariate multiple imputations are (1) data augmentation (Tanner and Wong, 1987), (2) sequential regression modeling (Raghunathan et al., 2001; Van Buuren et al., 2006), and (3) fully Bayesian modeling (Carrigan et al., 2007). The introductory works of Allison (2002), Enders (2010), or Graham (2012) focus on best practice in the use of data augmentation. The last author provides also a tutorial on how to handle missing data with SPSS. Van Buuren (2012) provides an excellent manual on the application of sequential regression modeling, also known, as imputation by chained equations. How to estimate missing values within a fully Bayesian framework by means of the software BUGS is explained by Lunn et al. (2012).

**Table 2:** **Amount of missing values in the analysis sample (*n* = 260)**.

	**Missing values (%)**
**PRE**	
Demographic and anamnestic data	0–17
Axis II diagnosis	13
Self-report (OQ-45)	2
**PROCESS**	
Self-report (OQ-45, HAQ-P)	12
Therapist-report of alliance (HAQ-T)	11
**POST**	
Self-report (OQ-45)	19

Our protocol can be basically applied to all three imputation procedures (essential parts of the program code can be found in the Supplementary Material). We generated the imputations following the second approach and using the functions from the R package mi (version 0.09–19; Su et al., 2011). Imputations of the longitudinal data were calculated using the normal linear regression. The application of this model requires the repeated measures to be organized in a wide format. Since the therapies analyzed have a varying number of measurement occasions, not all time points can be used as covariates. Therefore, we summarized the longitudinal data by the following set of common covariates:

- *Subject-specific intercepts and slopes*. The time-varying measures were summarized by solving, through the ordinary least square method, the equation *y_ij_* = β_0_ + β_1_*s_ij_* + *e_ij_* in which the score *y_ij_* of the subject *i* on occasion *j* on a single process scale (i.e., HAQ-P-H) was regressed on the session number *s_ij_*. Intercept β_0_ and slope β_1_ were used as predictors in the imputation models. Solely process measurements were used (i.e., pre-values were excluded from the computation).
- *Pre-values (OQ-45) and values from the last two process measurements (OQ-45, HAQ)*.

In addition the following subject-level covariates were taken in consideration in building the imputation equations: sociodemographic data, clinical history, Axis I and Axis II diagnoses, treatment orientation, number of sessions, time interval between last therapy session and post-assessment. Both the time-varying and the subject-level covariates built a pool of 110 potential predictors. Given the ratio of variables to cases, the inclusion of all predictors in the single regression equations increase the risk of multicollinearity. Therefore, we reduced the number of predictors in each equation using the following criteria:

- *Use of monotone data imputation, if applicable*. Scales, which are intended to be completed by the same person on the same occasion, contain possible missing values contemporaneously. Hence, we treated these scales with the monotone pattern imputation (Rubin, 1987, pp. 171–174; Van Buuren, 2012, p. 104).
- *Reducing the number of terms in models exhibiting, in the preliminary complete case analysis, any values higher than 10 of the variance inflation factor (VIF)*. In these models, single input variables that contribute to the multicollinearity are removed. Variables with the highest *p*-values are dropped first. In case there is lack of convergence of the imputation process, then the number of predictors in the model is reduced, until all VIF values fall below 5.

### Sensitivity assessment

Preliminary results with the current data indicated that, in the presence of MNAR, the amount of bias in estimates obtained from multiply imputed data depends on the composition of the observed sample, on the amount of missing data and on the difference between the true parameter value and the estimate obtained from the observed data. No rules of thumb can be formulated concerning the question as to in which situations multiple imputation delivers robust estimates, regardless of the underlying missing mechanism. Therefore, an approach to assess the accuracy and robustness of estimates obtained in different data sets is needed. The posterior predictive checking is a flexible approach from the Bayesian statistics used to test whether the model's predictions are consistent with the data (Lunn et al., 2012; Gelman et al., 2014). Gelman et al. (1998) combined the underlying principle of this technique with that of the cross-validation to test the performance of their imputation model with MAR as well as MNAR non-response. A further practical implementation of the posterior predictive checking as a diagnostic method for checking MI models was presented by He and Zaslavsky (2012) and extensively evaluated by Nguyen et al. (2015). Basically with these approaches, an imputed data set is taken as the basis for a simulation in which missing values are created and re-imputed. The imputed data sets are then compared with the one prior to deletion. Building upon this strategy we developed a sensitivity testing procedure, which additionally takes into consideration the concept of clinical significance. Clinical significance, that is, the determination of the proportions of improved and recovered cases, is a helpful complement to the pre-post effect size (Jacobson and Truax, 1991; Lambert et al., 2008). According to the manual (Lambert et al., 2004), a difference of 14 points in the OQ total score represents, at a confidence level of 95%, the minimal difference between two scores measured on different occasions that can be declared as a true change. Among the 210 complete cases in our sample, 132 had a post-value of at least 14 points lower than the respective pre-value and were considered *improved*. The remaining 78 cases were divided into 69 unchanged and 9 deteriorated patients who form in the following analyses the group labeled as *not improved*. The maximum bias to which the imputation procedure is exposed under an MNAR mechanism arises if the 50 cases with missing outcome are all either improved or not improved. The robustness of the imputation models in these scenarios will be tested using the following steps:

1. *Create multiple imputations under the MAR assumption (see preceding section)*.
2. *Take one imputed data set as the basis of a simulation*.
3. *Check the model fit to the data by examining the accuracy of MI under MAR conditions*.

Multiple imputation of values satisfying the MAR condition leads to unbiased estimates, unless assumptions of the applied model are violated. For instance, imputing normally distributed values by a linear model can lead to overestimating the central tendency in rating data that exhibit a floor effect (right skewed): this can be the case with ratings of the symptom distress collected at the end of the treatment. Substantial biases detected in MAR simulations are a sign that the imputation model is misspecified and needs to be revised before continuing the analyses (i.e., back to step 1). In our checks we simulated non-response with a probability based on outcome values observed at earlier time points (the basic principle for generating MAR data is described, e.g., by Van Buuren, 2012, p. 63). Possible biases were checked as described in paragraph 4.4.

4. *Test the robustness of the imputation results against MNAR*.

Let *b*, *c*, and *d* denote the observed number of improved cases, not improved cases and cases with missing outcomes in the original sample (before the multiple imputation). In our example we had *b* = 132, *c* = 78, and *d* = 50. Further let γ be the pre-post effect size (Becker, 1988) in the population and $\hat{\gamma }$ its estimate from a sample.

4.1. Draw with replacement from the reference data *b* improved and *c* not improved cases. This step reproduces the composition of the subsample with observed outcome data.
4.2. Draw with replacement from the reference data *d* not improved cases. Delete the outcome values of these cases.
4.3. Carry out the imputation procedure as in step 1 with this new sample consisting of *b* + *c* + *d* cases.

We repeated steps 4.1–4.3 1000 times. The average effect size over the 1000 samples before deletion was taken as the “true” effect size γ.

4.4. *Assess the bias by comparing the estimates obtained after imputation with the “true” parameter value*. Possible evaluation criteria are the percentage bias or the standardized bias (Burton et al., 2006). If the amount of bias is substantial, continue with steps 5 and 6. In our example we took a percentage bias of maximum 5% (Demirtas et al., 2008) as the criterion of robustness $100\%\cdot (\bar{\hat{\gamma }}-\gamma )/\gamma \leq 5\%$.

5. *Impute under an MNAR assumption*.

In the presence of substantial positive bias, outcome analyses should also be carried out under the assumption that subjects with missing outcome data have higher scores than respondents with the same covariates values. Rubin (1987, pp. 203–204) described simple pattern mixture models and selection models for the imputation of MNAR.

5.1. Delta-adjustment

Let Y, X, and M denote the outcome score, the covariates, and the missingness indicator, respectively (*M* = 0 if the score is observed, otherwise *M* = 1).

If only the mean of Y is of interest, then the following simple pattern mixture model can be applied; *E*(*Y*|*X*, *M* = 1) = *E*(*Y*|*X*, *M* = 0) + Δ, which adds a constant value to values imputed from an MAR model in order to get MNAR imputations (National Research Council, 2010). The bias assessment from step 3.4 can help in selecting values for Δ.

5.2. Selection model approach.

This approach can be applied in creating imputations under the assumption that the probability of non-response is proportional to the outcome score, i.e., the higher the level of dysfunctionality, the higher the probability to miss the post-assessment. We applied the following procedure, derived from Rubin (1987), to create these kinds of MNAR imputations:

5.2.1. For each value to be imputed generate 10 imputations using the procedure from step 1. The imputed values sorted in ascending order form the vector v.
5.2.2. Draw 10 uniform random numbers between 0 and 0.2. These values sorted in ascending order form the probability vector *p*. The association between M and Y can be modified by choosing either different boundaries of the uniform distribution or functions describing mechanisms other than MNAR-linear.
5.2.3. Draw from *v* weighted by *p* the definitive value for the MNAR imputation.

This strategy assumes that observed and missing values originate from a common distribution, but that unobserved values occupy higher percentiles than observed values.

6. *Compare estimates from the MAR and MNAR analyses*.

Altogether, the following four simulation trials were conducted:

- MAR1: The sampling procedure consisted of drawing with replacement 260 cases from the basis data set. The post-assessment values of 50 cases, still not improved at the last process measurement, were deleted.
- MAR2: Same sampling procedure as MAR1. The values of the last process measurement of 50 cases, still not improved at the penultimate process measurement, were deleted. The outcomes were evaluated at the last process measurement (post-assessment values were ignored).
- MNAR1: The sampling procedure was executed with *b* = 132, *c* = 78, and *d* = 50. The post-assessment values of 50 not improved cases were deleted.
- MNAR2: The sampling procedure was executed with *b* = 132, *c* = 78 but with *d* = 50 improved cases. The post-assessment values of 50 improved cases were deleted.

In each of the four simulation trials the analyses were additionally carried out using two simple techniques: complete case analysis (CC, others known as listwise deletion) and last-observation-carried-forward analysis (LOCF). For each estimator point estimates and confidence intervals based on percentiles (Efron and Tibshirani, 1994) were calculated.

### Assessing the predictive value of OQ-45 and HAQ

The analyses focused on two main questions:

- Can the initial improvement and the initial alliance quality predict the overall improvement (Model A) and the type of termination (Model B)? The initial improvement was defined as the difference between OQ total pre and OQ total at 5th session. HAQ-P and HAQ-T scores at 5th session were taken as indicators of the initial alliance quality.
- Can the HAQ scores measured during the therapy on occasion *t_i_* predict the subsequent improvement (Model C)? The difference between OQ total on occasion *t_i_* and OQ total on occasion *t*_*i* + 1_ was taken as subsequent improvement.

These analyses were conducted using mixed models with random intercepts. Models A and B had a nested three level structure with patients (*n* = 260) within therapists (*n* = 70) within treatment approaches (*n* = 10), whereas in Model C measurement occasions (*n* = 2032) were nested within patients (*n* = 260). Continuous independent (input) variables were rescaled by centering and dividing by two standard deviations so as to make their regression coefficients comparable to those of binary predictors (Gelman, 2008). Nested models based on multiply imputed data sets were compared using the likelihood ratio based procedure proposed by Meng and Rubin (1992). Model coefficients and contrasts were tested at a significance level of 5%.

## Results

### Robustness of the imputation procedure

**Figure 1:**
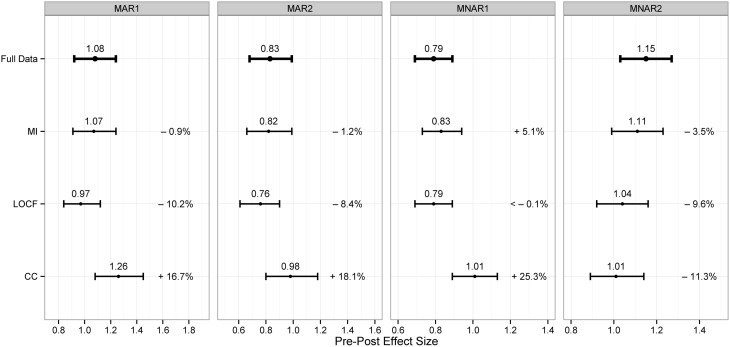
**Results of the simulations: Point estimates, CIs 95% and percentage biases**.

The simulation results are summarized in Figure 1. First of all, it is apparent that already with a missing rate just under 20% CC produced in all settings point estimates affected by an amount of bias of more than 10%. As expected LOCF gave conservative effectiveness estimates. It provided an unbiased point estimate in the worst-case scenario MNAR1. However, in both MAR conditions in which subjects with a modest outcome dropped out, this method led to a substantial under-estimation of more than 5%. MI provided on average more accurate parameter estimates than the other approaches. In both MAR simulations the MI point estimates exhibited a small bias due to departure from normality of the pre-post differences in the group with no improvement.

Since the percentage bias exceeded 5% in the worst-case simulation (MNAR1), missing values were re-imputed under the MNAR assumption as described in step 5. On average, the pre-post difference of the 50 cases with missing outcome data was overestimated by 4.5 points. This deviance was taken as Δ for the adjustment model. The selection model was conducted under the assumption of an MNAR-linear missingness. Both correction approaches reduced the bias to under 1% in the MNAR1 simulations.

In the original data set the effect size estimated by the delta-adjustment and the selection model were 1.00 and 1.02, respectively and did not differ substantially from the 1.08 obtained under the MAR assumption.

The re-analysis, under the MNAR assumption, of the models presented in Tables 3, 4 led essentially to the same regression coefficients as under the MAR approach. Therefore, the impact of MNAR data on the analysis conclusion was negligible.

**Table 3:** **Mixed models for the prediction of the overall improvement (Gaussian) and of the propensity to drop out (binomial)**.

**Covariates**		**Model A**			**Model B**		
		**OQ total pre-post**			**Dropout**		
		**Coefficients**	**SE**	***p***	**Coefficients**	**SE**	***p***
(Intercept)		33.51	4.36	<0.001	1.84	0.57	0.001
Dropout		**−18.19**	**3.36**	**<0.001**	−	−	−
Axis-I principal diagnosis	Anxiety	−0.26	3.2	0.937	0.12	0.52	0.813
	Adjustment	5.91	3.91	0.133	−0.47	0.72	0.514
	Other	−2.07	5.24	0.694	0.60	0.68	0.376
	None	2.88	5.27	0.587	0.54	0.82	0.506
Axis-I comorbidity		−0.44	2.25	0.847	−0.11	0.25	0.659
Axis-I lifetime		**−5.53**	**2.75**	**0.047**	−0.12	0.38	0.758
Axis-II: One or more PD		−5.20	2.81	0.068	−0.07	0.38	0.850
Treatments in the last 2 years		−**6.07**	**2.84**	**0.035**	0.26	0.39	0.505
Level of education: Low		1.78	4.64	0.703	0.97	0.56	0.080
Level of education: High		−3.87	2.94	0.194	0.31	0.39	0.431
OQ total: Pre value		**25.28**	**3.46**	**<0.001**	0.73	0.52	0.159
Initial improvement (pre-5th session)		**6.40**	**2.80**	**0.024**	−0.22	0.42	0.596
HAQ-P: 5th session		**7.94**	**2.88**	**0.007**	0.37	0.4	0.351
HAQ-T: 5th session		−0.98	2.51	0.696	−**1.41**	**0.40**	**<0.001**
		**Random effects**			**Random effects**		
		$\hat{\sigma }$_m_	$\hat{\sigma }$_t_	$\hat{\sigma }$_resid_	$\hat{\sigma }$_m_	$\hat{\sigma }$_t_	
		3.95	1.04	17.45	<0.01		0.41	

*Reference categories: Axis-I principal diagnosis = affective, level of education = middle. Variance components: $\hat{\sigma }$_t_ = therapist, $\hat{\sigma }$_m_ = therapy method*.

*Significant coefficients are highlighted in bold*.

**Table 4:** **Prediction of the subsequent improvement using a mixed model based on process measurements (PM)**.

**Covariates**	**Model C**		
	**Subsequent improvement OQ total**		
	**Coefficients**	**SE**	***p***
(Intercept)	2.33	0.76	0.002
OQ total	24.73	1.29	<0.001
HAQ-P-H	−1.86	1.17	0.113
Penultimate PM	4.82	1.04	<0.001
Last PM	8.64	1.22	<0.001
OQ total × penultimate PM	0.02	2.27	0.993
OQ total × last PM	3.81	2.54	0.135
HAQ-P-H × penultimate PM	**8.08**	**2.62**	**0.002**
HAQ-P-H × last PM	**7.19**	**2.53**	**0.005**
	**Random effects**		
	$\hat{\sigma }$_patient_	$\hat{\sigma }$_residual_	
	9.93		12.82	

*Significant coefficients of the therapeutic alliance are highlighted in bold*.

### Predictive value of the OQ-45 and HAQ ratings

The capability of the imputation model in reducing the bias under MNAR conditions argues for the predictive value of the OQ-45 and HAQ ratings. The scores of the OQ total and of the HAQ scales collected in the two last process measurements are the best predictors of the score at the end of the treatment (*R*^2^ = 0.63). By adding as predictors the scores collected at the third last process measurement did not incremented significantly the proportion of explained outcome variance.

**Figure 2:**
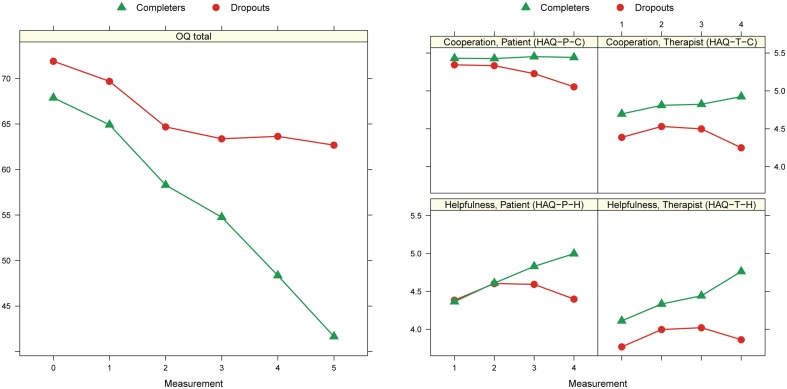
**Marginal means at different time points: 0 = pre, 1 = 1st process measurement (PM), 2 = intermediate PMs, 3 = penultimate PM, 4 = last PM, 5 = post**. Results from mixed models with random intercepts for patients and fixed effects for time occasion and kind of termination.

Figure 2 shows the treatment progress modeled for the OQ total scale and each of the HAQ subscales separately. The OQ total score indicate a stagnation of the progress among the dropouts in the last months before the discontinuation. Dropouts had at pre-assessment a higher OQ total score, although not statistically significant, than completers. Among the alliance ratings the HAQ-T-C and HAQ-T-H scores differentiated the most between dropouts and completers. Therapists rated already at the 5th session the alliance quality of dropouts significantly lower than that of completers. In contrast alliance ratings delivered by the patients, i.e., HAQ-P-C, and HAQ-P-H, in the initial treatment phase did not show any significant difference. At the last process measurement dropouts had significant lower scores on all four alliance subscales than completers.

With Models A and B the predictive value of the initial ratings together with other relevant covariates was tested (Table 3). The pre-post-difference of the OQ total score was the most related to the OQ total score itself at pre-assessment. Including this scale in the regression equation make the current principal diagnoses (Axis I and II) redundant as outcome predictors. On the contrary, data on history of psychological illness has a predictive value: patients with lifetime disorders or who recently already received a mental health treatment attain a smaller pre-post difference than patients without these characteristics. It is worth to mention that patients with low educational level do not reach less improvement than patients with higher education. Both, the initial improvement and the initial therapeutic alliance judged by the patient contribute significantly to predict the extent of pre-post change. This positive association remains if the dropout indicator is removed from the equation. Removing HAQ-P from the equation decreases the model fit significantly [*F*_(1, 247.2)_ = 7.7, *p* = 0.006]. Concerning the propensity to dropout, the only significant predictor is the initial alliance quality judged by the therapists (Model B).

Model C tests whether HAQ-P-H, which is the alliance subscale the most sensitive to change, can, with its collected scores during the therapy, predict subsequent improvement (Table 4). When controlling for OQ total scores, HAQ-P-H scores in the initial treatment phase do not predict subsequent improvement. On the contrary, in the final phase the predictive contribution of this scale is significant, i.e., the higher is judged the helpfulness the larger is the expected subsequent improvement. Removing HAQ-P-H from the equation decreases the model fit significantly [*F*_(3, 150.9)_ = 4.7, *p* = 0.004].

## Discussion

The present paper proposes a strategy to carry out MI with inclusive sensitivity analysis in quality assurance assessments or in non-randomized effectiveness studies in the field of mental health treatments. Longitudinal data collected in this kind of projects are, independently of their completeness, irregular, in the sense that, the number of measurement occasions and the overall monitored period is varying from subject to subject. Therefore, analysis methods considering MAR data, such as mixed models or MI, need to be adapted in order to adequately consider this irregularity. We proposed to summarize longitudinal data, which are used as predictors in the imputation of the missing outcome data at the end of the treatment, in two different ways: (1) as subject-specific intercepts and slopes, (2) by taking only the pre-values and the values from the two last process measurements. The first type of predictors can be useful in the imputation of missing diagnostic data at intake. Obviously using both type of variables contemporaneously create a certain redundancy, therefore it is important to check the multicollinearity of the imputation models in advance.

We tested the robustness of our MI models with a simulation procedure based on the Bayesian technique of posterior predictive checking.

The proposed approach can be used for the following purpose:

1. Quantifying the degree of bias introduced by MNAR mechanisms in a possible worst reasonable case scenario (MNAR simulations).
2. Comparing the performance of different analysis methods for dealing with missing data (MAR as well MNAR simulations).
3. Detecting the influence of possible violations to the regression model assumptions such as lack of normality (MAR simulations).

MI, which can be used as primary analysis method in outcome evaluations, leads to unbiased results under the MAR assumption. However, since it is likely that some missing data are MNAR, these primary results should be compared with results obtained under MNAR assumptions. In order to test the impact of MNAR mechanisms and, if necessary, to obtain indications on defining a suitable model for an alternative outcome estimation under MNAR conditions, we have suggested a procedure, in which the maximal degree of bias under a *clinically plausible* worst-case scenario is estimated by simulations. Our procedure integrates the concept of clinical significance used in the evaluation of the outcome quality of psychotherapies. Clinical significance consists in classifying patient outcome into four categories: improved, remitted, unchanged, and deteriorated. Due to the small number of deteriorated cases, we simplified in our example the categorization in a positive outcome (improved) and a negative outcome (not improved) (Lutz et al., 2006), but the application of all four categories is straightforward.

The simulation procedure allows, taking account of the composition of the observed data, to answer the question: How accurate are the estimates when subjects with missing outcome data have all a negative outcome? In line with Graham (2012), who favors the focus on the practical significance of the bias when the performance of MI is tested, we based our judgment on the percentage bias in estimating pre-post effect sizes. Another accuracy measure widely used is the standardized bias, which divides the bias by the standard error of the parameter. Collins et al. (2001) report that a standardized bias larger than 40% substantially lowers the confidence interval coverage and is therefore of practical concern. Graham (2012) states that the percentage bias is not sensitive to sample size but with large parameters can become statistically significant. He suggests using both measures with SB < 40% and PB < 10% indicating an acceptable bias.

With the simulation trials we also took the opportunity to compare the performance of MI with that of two simple methods: CC and LOCF. This comparison addresses the question of cost-benefit ratio: “Is it worth carrying out multiple imputations? Can satisfactory results also be obtained with simple approaches?” Our results shown that already with a missing rate of about 20% CC estimates are affected by a percentage bias greater than 10%. Users not familiar with the taxonomy of missing data mechanisms often ask about the maximum percentage of missing values with only a negligible effect on results obtained with CC. According to the literature this threshold is at about 10% (Barzi and Woodward, 2004; Kristman et al., 2004; Wood et al., 2004), a rate that is easily surpassed in observational studies.

The other simple technique, LOCF, proved to be particularly conservative and led often to a substantial under-estimation of the pre-post effect in the whole sample. In turn, MI demonstrated a better capability to discriminate between improved and not improved subjects. This kind of flexibility is important as not every missing outcome measurement is the consequence of treatment failures; withdrawing the assessment can be the consequence of previous negative experiences with the assessment itself.

In order to obtain with MI a robust outcome estimate at the end of the treatment, repeated measurements of relevant predictors not only of missingness but also of outcome are essential. Published simulation studies demonstrated that including auxiliary variables, which correlate with outcome but not necessarily with missingness, can considerably reduce the bias caused by MNAR (Collins et al., 2001; Demirtas, 2004). Obviously collecting an extensive number of clinical data every week would grant the best robustness against MNAR mechanism, but such an additional task augments the administrative expense and reduces the commitment of patients and therapists. Although not systematically investigated, this study addressed the question on required frequency and range of data collection in order to get robust imputations. In our project, data were collected after each 5th therapy session; at the beginning of the therapy the frequency were usually 1 session per week and it lowered during treatment progress, so that the mean interval between the last three measurements was 14 and 17 weeks, respectively. With this frequency and with a missing rate of about 20% MI yielded good results under MNAR conditions reducing the bias under 10%. With our data, however, we had to renounce including incomplete therapies with less than 10 sessions (9%), since the MI procedure requires longitudinal data with at least two measurement occasions. Thus, the system can be improved by increasing the frequency of measurement at the beginning of the treatment.

The question concerning the range of data collection refers particularly to the benefit of an additional measurement of the therapeutic alliance for the robustness of MI results. There is no doubt about the importance of the therapeutic alliance for the success of psychotherapy. What remains unclear is whether alliance questionnaires really ascertain information that is not already delivered by questionnaires measuring the patient's progress. The predictive value of the HAQ was therefore tested with different regression models, in which additionally the OQ-45 score was controlled. The results show that the initial alliance quality rated by the patient can predict the overall improvement, whereas the initial rating delivered by the therapist can predict the discontinuation decided by the patient. The latter result is in contrast with previous research related to the OQ-45, in which the capability of therapist in predicting the outcome of their patients, without feedback from a routine outcome measurement, was tested (Hannan et al., 2005). The authors of the study concluded that “therapists tend to overpredict improvement and fail to recognize clients who worsen during therapy” (Hannan et al., 2005, pp. 161). Our results did not indeed prove that therapists can accurately predict the degree of change in their patients, but that they can predict difficult treatment courses that are at risk of drop out.

Another positive result from our analyses concerns the capability of the Helpfulness score rated by the patient during the final therapy phase to improve the prediction of subsequent improvement. The HAQ has often been criticized because it does not separate between alliance and symptomatic improvement (Luborsky et al., 1996). This characteristic seems however an advantage when it comes to predict the outcome.

All in all, we can recommend repeated measurements with the OQ-45 and the HAQ in the evaluation of outpatient psychotherapy. Collected data do not only serve improvement of accuracy of the outcome estimate, they can also be used as feedback instruments for therapists and patients.

### Conflict of interest statement

The authors declare that the research was conducted in the absence of any commercial or financial relationships that could be construed as a potential conflict of interest.
