# Supplementary material for: Sensitivity analysis in multiple imputation in effectiveness studies of psychotherapy
Source: Front Psychol. 2015 Jul 27;6:1042. doi: 10.3389/fpsyg.2015.01042 (PMC4515885; doi:10.3389/fpsyg.2015.01042)
Supplement: Supplementary file 1 [file DataSheet1.PDF]

Supplementary Material: Sensitivity analysis in multiple imputation in effectiveness studies of psychotherapy

Aureliano Crameri*, Agnes von Wyl, Margit Koemeda, Peter Schulthess and Volker Tschuschke

*Correspondence:
Aureliano Crameri:
aureliano.crameri@zhaw.ch

1 SUPPLEMENTARY DATA

```
##########################################################################################
#   Program code # 1: How to summarize irregular longitudinal data
#   The data frames in Supplementary Tables 1 and 2 are taken as input.
#   The final data frame is displayed in Supplementary Table 3.
##########################################################################################

library(reshape)
# Keeping only the last two process measurements
    penultimate <- long[long$end == "penultimate", c("ID","HAQ","OQ")]
    penultimate <- rename(penultimate, c("HAQ" = "HAQ_pu","OQ" = "OQ_pu"))
    ultimate <- long[long$end == "ultimate", c("ID","HAQ","OQ")]
    ultimate <- rename(ultimate, c("HAQ" = "HAQ_u","OQ" = "OQ_u"))
    wide.s <- merge( merge(wide[ , c("ID", "AxisII", "treatment_2y", "duration", "n_sessions",
                     "OQ_pre", "OQ_post")], penultimate, all.y = T, by = "ID"),
                     ultimate, all.y = T, by = "ID" )

# Summarizing all process measurements as individual intercepts and slopes (OLS)
    coeffOQ <- t(sapply(unique(long$ID), function(x) lm(OQ ~ session,
                  subset = (ID == x), data = long)$coefficients))
    rownames(coeffOQ) <- unique(long$ID); colnames(coeffOQ) <- c("OQ_incpt","OQ_slope")
    coeffHAQ <- t(sapply(unique(long$ID), function(x) lm(HAQ ~ session,
                  subset = (ID == x), data = long)$coefficients))
    rownames(coeffHAQ) <- unique(long$ID); colnames(coeffHAQ) <- c("HAQ_incpt","HAQ_slope")

# Final data frame
    wide.s <- merge( merge(wide.s, coeffOQ, by.x= "ID", by.y = "row.names"), coeffHAQ,
                     by.x = "ID", by.y = "row.names")
```

```
################################################################################
# Program code #2: Imputation functions
# The following functions are abbreviated and modified versions
# of those in the package mi 0.09-19 created by Gelman et al.
################################################################################

# The Original imputation functions can be installed as follows: #
# packageurl_1 <- "http://cran.r-project.org/src/contrib/Archive/arm/arm_1.7-07.tar.gz"
# packageurl_2 <- "http://cran.r-project.org/src/contrib/Archive/mi/mi_0.09-19.tar.gz"
# install.packages(c(packageurl_1, packageurl_1), repos=NULL, type="source")

# Overview:
#----------------#----------------------------------#----------------------------------------#
# Function name  # Response variable                # Model                                  #
# ---------------#----------------------------------#----------------------------------------#
# mi.continuous  # continuous, normally distributed # gaussian(link = "identity")            #
# mi.pmm         # continuous                       # predicitve mean matching               #
# mi.binary      # dichotomous                      # binomial(link = "logit")               #
# mi.count       # count                            # quasipoisson(link = "log")             #
# mi.polr        # ordered categorical              # proportional odds logistic regression  #
# mi.categorical # unordered categorical            # multinomial                            #
# ---------------#----------------------------------#----------------------------------------#

mi.continuous <- function (formula, data, prob.func = NULL, delta = NULL, ...)
{
    if (!is.null(delta) & !is.null(prob.func))
        stop(message = "Delta-adjustment and selection model cannot be performed at the same time.")
    DV <- as.character( formula[[2]] )
    Y <- data[ ,DV]
    missing.index <- is.na(Y)
    n.mis <- sum(missing.index)

    # Model
        bglm.imp <- bayesglm(formula = formula, data = data, family = gaussian,
                             drop.unused.levels = FALSE, Warning = FALSE)
        mf <- model_frame(bglm.imp, data[missing.index, , drop = FALSE])

    # Drawing parameter values from the posterior distribution
        sim.bglm.imp <- sim(bglm.imp, 1)
        sim.coef <- sim.bglm.imp@coef
        sim.sigma <- sim.bglm.imp@sigma
        y_hat <- as.matrix(tcrossprod(mf, sim.coef))
```

```
    # Drawing imputations from the posterior predictive distribution
        if (is.null(prob.func))
        {
            if (is.null(delta))
            { # MAR imputations (default)
                random.pred <- rnorm( n.mis, y_hat, sim.sigma )
                } else
                { # MNAR imputations by delta-adjustment
                  # (subsection Sensitivity Assessment, paragraph 5.1)
                    random.pred <- rnorm( n.mis, y_hat, sim.sigma ) + delta
                }
        } else
        { # MNAR imputations under a selection model
          # (subsection Sensitivity Assessment, paragraph 5.2)
            expr=parse(text=prob.func); n.draw <- length(eval(expr))
            random.matrix <- matrix(replicate(n.draw,
                                    rnorm(n.mis, y_hat, sim.sigma)), nrow=n.mis) # vector v
            random.matrix  <- t(apply(random.matrix,1,sort))
            prob.matrix <- t(sapply(1:n.mis, function(x)sort(eval(expr)))) # vector p
            random.pred <-  sapply(1:n.mis, function(x, y=random.matrix, z=prob.matrix)
                                                        sample(y[x,],size=1, prob=z[x,]))
        }
    names(random.pred) <- rownames( data[ missing.index, ] )
    return(random.pred)
}

mi.pmm <- function (formula, data, ...)
{
    DV <- as.character(formula[[2]])
    Y <- data[ ,DV]
    missing.index <- is.na(Y)
    n.mis <- sum(missing.index)
    bglm.imp <- bayesglm(formula = formula, data = data, family = gaussian,
                         drop.unused.levels = FALSE, Warning = FALSE, ...)
    mf <- model_frame(bglm.imp, data[, , drop = FALSE])
    sim.bglm.imp <- sim(bglm.imp, 1)
    sim.coef <- sim.bglm.imp@coef
    sim.sigma <- sim.bglm.imp@sigma
    yhat <- as.matrix(tcrossprod(mf, sim.coef))
    random.pred   <- apply( as.array( yhat[missing.index] ), 1, mi.pmm.match,
                            yhat=yhat[!missing.index], Y=Y[!missing.index] )
    names(random.pred) <- rownames(data[missing.index,])
    return(random.pred)
}
```

```
mi.binary <- function (formula, data, ...)
{
    DV <- as.character(formula[[2]])
    Y <- data[ ,DV]
    missing.index <- is.na(Y)
    n.mis <- sum(missing.index)

    # recode DV in 0/1 #
        y.levels <- if (is.numeric(Y)) { sort(unique(Y)) }
        else if (is.logical(Y)) { c(FALSE, TRUE) }
        else { levels(factor(Y)) }
        Y <- recode(Y, paste("'", y.levels, "'=", c(0, 1), sep = "",
        collapse = "; "))

    bglm.imp <- bayesglm(formula = formula, data = data,
                         family = binomial(link = "logit"),
                         drop.unused.levels = FALSE, Warning = FALSE, ...)
    mf <- model_frame(bglm.imp, data[missing.index, , drop = FALSE])
    sim.coef <- sim(bglm.imp, 1)@coef
    prob.pred <- invlogit(as.matrix(tcrossprod(mf, sim.coef)))
    random.temp <- rbinom(n.mis, 1, prob.pred)
    random.pred <- random.temp
    random.pred[random.temp == 0] <-  y.levels[1]
    random.pred[random.temp == 1] <-  y.levels[2]
    names(random.pred) <-   rownames(data[missing.index,])
    if (is.logical(y.levels)) { random.pred <- as.logical(random.pred) }
    return(random.pred)
    on.exit(rm(bglm.imp))
}

mi.polr <- function (formula, data, ...)
{
    DV <- as.character(formula[[2]])
    missing.index <- is.na(data[ ,DV])
    n.mis <- sum(missing.index)
    bplr.imp <- bayespolr(formula = formula, data = data, start = 0,
                          method = c("logistic"), drop.unused.levels = FALSE)
    expect.prob <- predict(bplr.imp, newdata = data, type = "probs")
    random.pred <- Rmultnm(n.mis, expect.prob[missing.index, ], 1:length(bplr.imp$lev))
    random.pred <- recode(random.pred, paste(1:length(bplr.imp$lev),
                          "='", bplr.imp$lev, "'", sep = "", collapse = ";"))
    names(random.pred) <- rownames(data[missing.index,])
    return(random.pred)
}
```

```
mi.count <- function (formula, data, ...)
{
    DV <- as.character(formula[[2]])
    missing.index <- is.na(data[ ,DV])
    n.mis <- sum(missing.index)

    bglm.imp <- bayesglm(formula = formula, data = data, family = quasipoisson,
                          drop.unused.levels = FALSE, Warning = FALSE, ...)

    dispersion <- summary(bglm.imp)$dispersion
    mf <- model_frame(bglm.imp, data[missing.index, , drop = FALSE])
    sim.coef <- sim(bglm.imp, 1)@coef
    lambda <- exp(as.matrix(tcrossprod(mf, sim.coef)))
    random.pred <- rpois.od(n = n.mis, lambda = lambda, dispersion = dispersion)
    names(random.pred) <- rownames(data[missing.index,])
    return(random.pred)
}

mi.categorical <- function (formula, data, ...)
{
    DV <- as.character(formula[[2]])
    Y <- data[ ,DV]
    missing.index <- is.na(Y)
    n.mis <- sum(missing.index)

    lm.cat.imp <- multinom(formula = formula, data = data, trace=FALSE, ...)
    deter.prob <- predict(lm.cat.imp, newdata = data, type = "p")
    y.cat <- levels(factor(Y))
    y.ncat <- length(y.cat)
    if (length(y.cat) <= 2) {
        stop(message = "Categories must be more than 2.")
    }
    random.pred <- Rmultnm(n.mis, deter.prob[missing.index, ], 1:y.ncat)
    random.pred <- recode(random.pred, paste(1:y.ncat, "='",
                   y.cat, "'", sep = "", collapse = ";"))
    names(random.pred) <- rownames(data[missing.index,])
    return(random.pred)
}
```

```
#--------------------------------
#  Subordinate functions
#--------------------------------

model_frame <- function(model, data)
{
    tt <- terms(model)
    Terms <- delete.response(tt)
    mf <- model.frame(Terms, data = data, xlev = model$xlevels)
    mf <- Matrix(model.matrix(Terms, mf, contrasts.arg = model$contrasts), sparse = TRUE)
    return(mf)
}

mi.pmm.match <- function(z, yhat=yhat, Y=Y)
{
    d <- abs( yhat - z )
    m <- Y[ d == min( d )]
    if ( length( m ) > 1 ) m <- sample( m, 1 )
    return( m )
}

Rmultnm <- function (n, prob.mat, category)
{
    y.imp <- NULL
    prob <- prob.mat * NA
    for (i in 1:n) { prob[i, ] <- rmultinom( 1, 1, prob.mat[i, ] ) }
    y.imp <- as.double(prob %*% category)
    return(y.imp)
}

rpois.od <- function (n, lambda, dispersion = 1)
{
    if (dispersion <= 1) {
        ans <- rpois(n, lambda)
    }
    else {
        B <- 1/(dispersion - 1)
        A <- lambda * B
        ans <- rnbinom(n, size = A, mu = lambda)
    }
    return(ans)
}
```

```
##########################################################################################
#
# Program code #3: MI by sequential regression modeling (subsection Imputation Procedure)
#
##########################################################################################

sequential.regression <- function( # Main function performing the iterations
                            s.data, # s.data is a single data frame containig missing values.
                            model.seq = model.seq, var.binary = NULL, var.categorical = NULL,
                            var.continuous = NULL,
                            var.continuous.delta=NULL, var.continuous.selection = NULL,
                            var.polr = NULL, var.count = NULL, var.pmm = NULL,
                            formulas, delta=NULL, prob.func=NULL,
                            boundaries = NULL,
                            n.imp, n.iter)
{
    all.var <- c(var.binary, var.categorical, var.continuous, var.continuous.delta,
                    var.continuous.selection, var.polr, var.count, var.pmm)
    var.quantitative <- c(var.continuous, var.continuous.selection, var.continuous.delta,
                          var.pmm, var.count)
    var.quantitative <- model.seq[model.seq %in% var.quantitative]
    var.qualitative <- c(var.binary, var.categorical, var.polr)
    var.qualitative <- model.seq[model.seq %in% var.qualitative]

    if (any(duplicated(all.var)))
        stop(message="More than one imputation model for the same variable")
    if (!all(model.seq %in% all.var))
        stop(message="One or more imputation models are missing")
    if (!is.null(var.continuous.delta) & is.null(delta))
        stop(message="Missing delta value(s)")
    if (!is.null(var.continuous.selection) & is.null(prob.func))
        stop(message="Missing probability function(s)")

    # Creating multiple data frames
        m.data <- list()
        for (i in 1:n.imp)
        {
            m.data[[i]] <- s.data
            # During the imputation process predictors must not have missing values.
            # Missing values are filled in by a preliminary hot-deck imputation.
            for (j in model.seq)
                {
                    missing <- rownames(s.data[is.na(s.data[,j]),])
                    observed <- rownames(s.data[!is.na(s.data[,j]),])
```

```
                m.data[[i]][missing,j] <-
                sample(s.data[observed,j], length(missing), replace=T)
            }
    }

# Quantities to be examined by trace plots
# Means and SDs for quantitative variables and proportions for qualitative variables respectively
l1 <- length(var.quantitative)
if (l1 > 0)
{
    means <- array(NA, dim=c(n.imp, n.iter, l1),
                dimnames=list(c(1:n.imp),c(1:n.iter), var.quantitative))
    sdev <- array(NA, dim=c(n.imp, n.iter, l1),
                dimnames=list(c(1:n.imp),c(1:n.iter), var.quantitative))
} else {means <- NULL; sdev <- NULL}

l1 <- length(var.qualitative)
if (l1 > 0)
{
    prop <- vector("list", l1)
    names(prop) <- var.qualitative
    for (i in 1:l1)
    {
        levels_i <- levels(s.data[, var.qualitative[i]])
        prop[[i]] <- array(NA, dim=c(n.imp, n.iter, length(levels_i)),
                        dimnames=list(c(1:n.imp),c(1:n.iter), levels_i))
    }
} else {prop <- NULL}

# Iterations
missing.indices <- lapply(model.seq, function(x) which(is.na(s.data[,x])))
names(missing.indices) <- model.seq
for (i in 1 : n.iter)
{
    cat(paste("\n\n", "Iteration:", i),"\n")
    for (j in 1:n.imp)
    {
        cat(paste("\n","Imputation:", j,"\n"))
        data1 <- m.data[[j]]
        for (k in model.seq)
        {
            missingness <- missing.indices[[k]]
            data1[missingness,k]<- NA # Deleting the values that were imputed in the last iteration
            imputed <- NULL
```

```
if (k %in% var.binary)
{
    cat(paste(k, "(mi.binary) ", sep=""))
    imputed <- mi.binary(formula=formulas[[k]], data=data1)
} else if (k %in% var.categorical)
{
    cat(paste(k, "(mi.categorical) ", sep=""))
    imputed <- mi.categorical(formula=formulas[[k]], data=data1)
} else if (k %in% var.continuous)
{
    cat(paste(k, "(mi.continuous) ", sep=""))
    imputed <- mi.continuous(formula=formulas[[k]], data=data1)
    imputed[imputed < boundaries[[k]][1]] <- boundaries[[k]][1]
    imputed[imputed > boundaries[[k]][2]] <- boundaries[[k]][2]
} else if (k %in% var.continuous.selection)
{
    cat(paste(k,"(mi.continuous.selection) ", sep=""))
    imputed <- mi.continuous(formula=formulas[[k]], data=data1,
                             prob.func=prob.func[[k]])
    imputed[imputed < boundaries[[k]][1]] <- boundaries[[k]][1]
    imputed[imputed > boundaries[[k]][2]] <- boundaries[[k]][2]
} else if (k %in% var.continuous.delta)
{
    cat(paste(k, "(mi.continuous.delta) ", sep=""))
    imputed <- mi.continuous(formula=formulas[[k]], data=data1,
                             delta=delta[[k]])
    imputed[imputed < boundaries[[k]][1]] <- boundaries[[k]][1]
    imputed[imputed > boundaries[[k]][2]] <- boundaries[[k]][2]
} else if (k %in% var.pmm)
{
    cat(paste(k, "(mi.pmm) ", sep=""))
    imputed <- mi.pmm(formula=formulas[[k]], data=data1)
} else if (k %in% var.count)
{
    cat(paste(k, "(mi.count) ", sep=""))
    imputed <- mi.count(formula=formulas[[k]], data=data1)
} else if (k %in% var.polr)
{
    cat(paste(k, "(mi.polr) ", sep=""))
    imputed <- mi.polr(formula=formulas[[k]], data=data1)
}
data1[missingness, k] <- imputed
if (k %in% var.qualitative)
{
    categories <- levels(data1[, k])
```

```
                    for (w in 1:length(categories))
                    {
                        prop[[k]][j,i, categories[w]] <- mean(imputed== categories[w])
                    }
                } else if (k %in% var.quantitative)
                {
                    means[j,i,k] <- mean(imputed)
                    sdev[j,i,k] <- sd(imputed)
                }
            }
            m.data[[j]] <- data1
        }
    }
    return(list(m.data = m.data, means = means, sdev = sdev, prop = prop))
}


pool.lm <- function(formula, m.list) # Pools coefficients provided by the function lm() applied on
                                     # multiply imputed data frames
{
    if (class(m.list)!="list") {
        stop(message = "Data frames must be organized as a list.")
    }
    m1 <- imputationList(m.list)
    model1 <- with(m1,{
                 attr(formula, ".Environment") <- environment()
                 lm(formula)
                })
    coeff1<-MIextract(model1,fun=coefficients)
    var1<-MIextract(model1, fun=vcov)
    pool1 <- MIcombine(coeff1,var1, df.complete=model1[[1]]$df.residual)
    return(pool1)
}

add.es <- function(data, pre, post) # Adds the pre-post effect size as a new variable
{                                   # in a single imputed data frame
        name <- paste("es_", strsplit(pre,"_")[[1]][1], sep="")
        data[, name] <- (data[, pre] - data[, post])/sd(data[, pre])
        return(data)
}
```

```
plot.quantitative <- function(means, sdev, n.missing=NULL) # Trace plot, which is based on
{                                                          # means and SDs, for checking
    for (i in attr(means, "dimnames")[[3]])                # the convergence to a stationary
    {                                                      # distribution
        t_m <- melt(means[,,i, drop=F])[,-3]
        t_sd <- melt(sdev[,,i, drop=F])[,-3]
        if (mean(t_m[,2])>1) {type_symbol <- "l"} else {type_symbol <- "p"}
        names(t_m) <- names(t_sd) <- c("imputation","iteration", i)
        print(xyplot(t_m[, i] ~ iteration, groups=imputation, data=t_m, type=type_symbol,
                     ylab=i, main=paste("Mean (n=", n.missing[i],")",sep="")))
        print(xyplot(t_sd[, i] ~ iteration, groups=imputation, data=t_sd, type=type_symbol,
                     ylab=i, main=paste("SD (n=", n.missing[i],")",sep="")))
    }
}

plot.qualitative <- function(prop, n.missing=NULL) # Trace plot based on proportions
{
    for (j in attr(prop, "names"))
        {
            for (i in attr(prop[[j]], "dimnames")[[3]])
            {
                t_p <- melt(prop[[j]][,, i,drop=F])[-3]
                if (mean(t_p[,2])>1) {type_symbol <- "l"} else {type_symbol <- "p"}
                names(t_p) <- c("imputation","iteration", i)
                print(xyplot(t_p[, i] ~ iteration, groups=imputation, data=t_p,
                      type=type_symbol, ylab=paste(j,": ",i,sep=""),
                      main=paste("Proportion (n=", n.missing[j],")",sep="")))
            }
        }
}


path <- "/Users/aure/Documents/chartaMacBook/Auswertungen/Outcome3"
setwd(path)
load("wide.s.RData")
n.iter <- 40 # Number of iterations
n.imp <- 10 # Number of imputations

# List of variables to be imputed
    var.binary <- c("treatment_2y")
    var.categorical <- c("AxisII")
    var.continuous <- c("OQ_pre", "HAQ_pu","OQ_pu","HAQ_u","OQ_u", "OQ_post")
    model.seq <- c("AxisII", "treatment_2y", "OQ_pre",
                   "HAQ_pu","OQ_pu","HAQ_u","OQ_u","OQ_post") # Sequential order of the variables
    n.missing <- sapply(model.seq, function(x) sum(is.na(wide.s[,x])))
```

```
# Regression equations
    formulas <- list()
    formulas$AxisII <- (AxisII ~ treatment_2y + n_sessions + duration + OQ_pre + OQ_post +
                        HAQ_pu + OQ_pu+ HAQ_u + OQ_u +
                        OQ_incpt + OQ_slope + HAQ_incpt + HAQ_slope)
    formulas$treatment_2y <- (treatment_2y ~ AxisII + n_sessions + duration + OQ_pre + OQ_post +
                                HAQ_pu + OQ_pu+ HAQ_u + OQ_u +
                                OQ_incpt + OQ_slope + HAQ_incpt + HAQ_slope)
    formulas$OQ_pre <- (OQ_pre ~ treatment_2y + AxisII + n_sessions + duration + OQ_post +
                        HAQ_pu + OQ_pu+ HAQ_u + OQ_u +
                        OQ_incpt + OQ_slope + HAQ_incpt + HAQ_slope)
    formulas$HAQ_pu <- (HAQ_pu ~ treatment_2y + AxisII + n_sessions + duration +
                         HAQ_u + OQ_u + OQ_pre + OQ_post +
                         OQ_incpt + OQ_slope + HAQ_incpt + HAQ_slope)
    formulas$OQ_pu <- (OQ_pu ~ treatment_2y + AxisII + n_sessions + duration + OQ_pre + OQ_post +
                        HAQ_pu + HAQ_u + OQ_u +
                        OQ_incpt + OQ_slope + HAQ_incpt + HAQ_slope)
    formulas$HAQ_u <- (HAQ_u ~ treatment_2y + AxisII + n_sessions + duration + OQ_pre + OQ_post +
                        OQ_pu + HAQ_pu  +
                        OQ_incpt + OQ_slope + HAQ_incpt + HAQ_slope)
    formulas$OQ_u <- (OQ_u ~  AxisII + n_sessions + duration + OQ_pre + OQ_post +
                        HAQ_pu + HAQ_pu + HAQ_u +
                        OQ_incpt + OQ_slope + HAQ_incpt + HAQ_slope)
    formulas$OQ_post <- (OQ_post ~ treatment_2y + AxisII + n_sessions + duration + OQ_pre  +
                        HAQ_pu + OQ_pu + HAQ_u + OQ_u +
                        OQ_incpt + OQ_slope + HAQ_incpt + HAQ_slope)

# Exploring the regression models in a CC analysis (Example OQ_post)
    model_cc <- lm(formulas[["OQ_post"]], data=wide.s)
    summary(model_cc)
    vif(model_cc) # Checking multicollinearity
    plot(model_cc) # Residual diagnostics

# Min / max values in rating scales: imputed values beyond these specified bounds will be replaced
# by the mininum and maximum respectively.
    boundaries <- list()
    boundaries$OQ_pre <- boundaries$OQ_post <- boundaries$OQ_pu <- boundaries$OQ_u <- c(0,180)
    boundaries$HAQ_pu <- boundaries$HAQ_u  <- c(1,6)
```

```
# Generating multiple imputations
    result <- sequential.regression(s.data = wide.s,
        model.seq = model.seq, var.binary = var.binary, var.categorical = var.categorical,
        var.continuous = var.continuous,
        formulas = formulas,
        boundaries = boundaries,
        n.imp = n.imp, n.iter = n.iter)
    m.data <- result[["m.data"]]
    save(m.data, file="m.data.RData")

# Trace plots for checking the convergence to a stationary distribution
    pdf(file="Trace_plot_quantitative.pdf")
        plot.quantitative(result[["means"]], result[["sdev"]], n.missing)
    dev.off()

    pdf(file="Trace_plot_qualitative.pdf")
        plot.qualitative(result[["prop"]], n.missing)
    dev.off()

# Analysis of multiply imputed data (Example: calculation of mean and effect size)
    pool.lm(OQ_post~1, m.data)$coefficients
    m.data <- lapply(m.data, add.es, "OQ_pre", "OQ_post")
    pool.lm(es_OQ~1, m.data)$coefficients
```

```
########################################################################################
#
# Program code #4: Sensitivity analysis by posterior predictive checking
# (subsection Sensitivity Assessment)
#
########################################################################################

    create.folder <- function(prefix, path)
    {
            time1 <- as.character(Sys.time())
            for (i in c(":"," ","-"))  time1 <- gsub(i,"_",time1)
            path_sim <- paste(path, "/",prefix,"-",time1, sep="")
            system(paste("mkdir -p ", path_sim,sep=""))
            return(path_sim)
    }

    model.seq  <- var.continuous <- c("OQ_post")
        n.imp <- 10
    n.iter <- 1 # There is only one variable missing in these examples; for univariate
                # imputations no iterations are required.
    n.sim <- 100
    d <- 50 # Number of cases with missing outcome data

#----------------------------------------------------------------------------------------------------
 # MAR simulations (subsection Sensitivity Assessment, paragraph 3)
 # Example: The post-assessment values of 50 cases, still not improved at the last process
 # measurement, were deleted.
#----------------------------------------------------------------------------------------------------
    setwd(path); load("m.data.RData")
    path_sim <- create.folder("MAR", path)
    time1 <- strsplit(path_sim,"-")[[1]][2]
    setwd(path_sim)

    for (i in 1:n.sim)
    {
        cat(paste("\n\n Simulation:",i,"\n"))
        folderName <- paste("s_", i,"_",time1, sep="")
        system(paste("mkdir -p ",path_sim,"/", folderName, sep=""))
        setwd(paste(path_sim,"/",folderName,sep=""))

        s.data <- m.data[[sample(length(m.data),1)]] # Chooses one imputed data frame at random
        s.data <- s.data[sample(rownames(s.data), replace=T), ] # Bootstrap
        rownames(s.data) <- NULL
        FD <- s.data # Full data
```

```
        save(FD, file=paste("FD_",i,"_",time1,".RData",sep=""))

        improved <- (s.data$OQ_pre-s.data$OQ_u)>=14
        deleted <- sample(rownames(s.data[!improved,]), d, replace=F)
        s.data[deleted, "OQ_post"] <- NA
        CC <- s.data # Data frame for CC analysis
        save(CC, file=paste("CC_",i,"_",time1,".RData",sep=""))

        result <- sequential.regression(s.data = s.data, model.seq = model.seq,
                    var.continuous = var.continuous,
                    formulas = formulas, boundaries = boundaries, n.imp = n.imp, n.iter = n.iter)
        save(result, file=paste("MI_",i,"_",time1,".RData",sep=""))
    }

#-------------------------------------------------------------------------------------------------
 # MNAR simulations (subsection Sensitivity Assessment, paragraph 4)
 # Example: The outcome of d=50 not improved cases is deleted. The subsample with observed outcome
 # data comprises b=132 improved and c=78 not improved cases.
#-------------------------------------------------------------------------------------------------
    setwd(path); load("m.data.RData")
    path_sim <- create.folder("MNAR", path)
    time1 <- strsplit(path_sim,"-")[[1]][2]
    setwd(path_sim)

    b <- 132; c <- 78 # Observed number of improved and not improved cases

    for (i in 1:n.sim)
    {
        cat(paste("\n\n Simulation:",i,"\n"))
        folderName <- paste("s_", i,"_",time1, sep="")
        system(paste("mkdir -p ",path_sim,"/", folderName, sep=""))
        setwd(paste(path_sim,"/",folderName,sep=""))

        s.data <- m.data[[sample(length(m.data),1)]] # Chooses at random one imputed data frame
        improved <- (s.data$OQ_pre-s.data$OQ_post)>=14
        obs_improved <- sample(rownames(s.data[improved,]), b, replace=T)
        obs_not_improved <- sample(rownames(s.data[!improved,]), c, replace=T)
        FD1 <- s.data[c(obs_improved,obs_not_improved),]
        miss_not_improved <- sample(rownames(s.data[!improved,]), d, replace=T)
        FD3 <- FD2 <- s.data[miss_not_improved,]
        FD <- rbind(FD1,FD2) # Data frame with fully observed data
        rownames(FD) <- NULL
        save(FD, file=paste("FD_",i,"_",time1,".RData",sep=""))

        FD3[,"OQ_post"] <- NA
```

```
        CC  <- rbind(FD1,FD3) # Data frame for a CC analysis
        rownames(CC) <- NULL
        save(CC, file=paste("CC_",i,"_",time1,".RData",sep=""))
        s.data <- CC

        result <- sequential.regression(s.data = s.data, model.seq = model.seq,
                    var.continuous = var.continuous,
                    formulas = formulas, boundaries = boundaries, n.imp = n.imp, n.iter = n.iter)
        save(result, file=paste("MI_",i,"_",time1,".RData",sep=""))
    }


#-----------------------------------------------------------------------------------------------------
 # Bias evaluation (subsection Sensitivity Assessment, paragraph 4.4)
#-----------------------------------------------------------------------------------------------------
    setwd(path_sim)

    # Reading the names of the folder and the files containing the output of the simulations
        l1 <- file.info(dir())
        l1 <- rownames(l1[l1$isdir,])
        name1 <- sub("s","",l1)

    # Creating matrices in which the estimates from each simulation are listed
        sample.es <- matrix(NA, nrow=length(l1), ncol=3) # Effect sizes
        colnames(sample.es) <- c("Full Data", "CC","MI")
        sample.mean <- sample.es # Outcome means
        miss.mean <- sample.es[,-2] # Outcome means of the subsamples with missing values

    # Calculating the estimates from each simulation
        for (i in 1: length(l1))
        {
            cat(paste(i," "))
            # Loading the files
                load(paste(path_sim,"/",l1[i],"/FD", name1[i],".RData",sep=""))
                load(paste(path_sim,"/",l1[i],"/CC", name1[i],".RData",sep=""))
                load(paste(path_sim,"/",l1[i],"/MI", name1[i],".RData",sep=""))
                missing <- is.na(CC$OQ_post)

            # Full data (reference data)
                sample.es[i,"Full Data"] <- mean(FD$OQ_pre-FD$OQ_post)/sd(FD$OQ_pre)
                sample.mean[i,"Full Data"] <- mean(FD$OQ_post)
                miss.mean[i,"Full Data"] <- mean(FD[missing,"OQ_post"])

            # CC
                sample.es[i,"CC"] <- mean(CC$OQ_pre-CC$OQ_post, na.rm=T)/sd(CC$OQ_pre, na.rm=T)
```

```
            sample.mean[i,"CC"] <- mean(CC$OQ_post, na.rm=T)

        # MI
            m.data <- result[["m.data"]]
            m.data <- lapply(m.data, add.es, "OQ_pre", "OQ_post")
            sample.es[i,"MI"] <- pool.lm(es_OQ~1, m.data)$coefficients
            sample.mean[i,"MI"] <- pool.lm(OQ_post~1, m.data)$coefficients
            mm.data <- lapply(m.data, function(x) x[missing,])
            miss.mean[i,"MI"] <- pool.lm(OQ_post~1, mm.data)$coefficients
    }

    # Summary
        means <- apply(sample.mean,2,mean)
        es <- apply(sample.es,2,mean)
        print(relbias.mean <- sapply(means, function(x) (x-means[1])/means[1]))
        print(relbias.es <- sapply(es, function(x) (x-es[1])/es[1]))
        print(delta <-  mean(miss.mean[,"Full Data"]) - mean(miss.mean[,"MI"]))


#-------------------------------------------------------------------------------------------------
 #  Simple MNAR models (subsection Sensitivity Assessment, paragraph 5)
#-------------------------------------------------------------------------------------------------

    var.binary <- c("treatment_2y")
    var.categorical <- c("AxisII")
    var.continuous <- c("OQ_pre", "HAQ_pu","OQ_pu","HAQ_u","OQ_u")
    model.seq <- c("AxisII", "treatment_2y", "OQ_pre",
                "HAQ_pu","OQ_pu","HAQ_u","OQ_u","OQ_post")
    n.imp <- 10; n.iter <- 40

    # Delta adjustment
        var.continuous.delta <- c("OQ_post")
        delta <- list(); delta$OQ_post <- 8
        result <- sequential.regression(s.data = wide.s,
            model.seq = model.seq, var.binary = var.binary, var.categorical = var.categorical,
            var.continuous = var.continuous,
            var.continuous.delta = var.continuous.delta, delta=delta,
            formulas = formulas,
            boundaries = boundaries,
            n.imp = n.imp, n.iter = n.iter)
        m.data <- result[["m.data"]]
        setwd(path); save(m.data, file="m.data.delta.RData")
        m.data <- lapply(m.data, add.es, "OQ_pre", "OQ_post")
        pool.lm(es_OQ~1, m.data)$coefficients
        pool.lm(OQ_post~1, m.data)$coefficients
```

```
    pdf(file="Trace_plot_quantitative_delta.pdf")
        plot.quantitative(result[["means"]], result[["sdev"]], n.missing)
    dev.off()

# Selection model
    var.continuous.selection <- c("OQ_post")
    prob.func <- list(); prob.func$OQ_post <- "runif(10, 0, 0.2)"
    result <- sequential.regression(s.data = wide.s,
        model.seq = model.seq, var.binary = var.binary, var.categorical = var.categorical,
        var.continuous = var.continuous,
        var.continuous.selection = var.continuous.selection, prob.func = prob.func,
        formulas = formulas,
        boundaries = boundaries,
        n.imp = n.imp, n.iter = n.iter)
    m.data <- result[["m.data"]]
    setwd(path); save(m.data, file="m.data.selection.RData")
    m.data <- lapply(m.data, add.es, "OQ_pre", "OQ_post")
    pool.lm(es_OQ~1, m.data)$coefficients
    pool.lm(OQ_post~1, m.data)$coefficients
    pdf(file="Trace_plot_quantitative_selection.pdf")
        plot.quantitative(result[["means"]], result[["sdev"]], n.missing)
    dev.off()
```

2 SUPPLEMENTARY TABLES

Supplementary Table 1. Irregular longitudinal data arranged in long format.

← subject-level variables →					← time-varying variables →			
ID	AxisII	treatment_2y	duration	n_sessions	session	end	HAQ	OQ
57	NA	no	265	12	5	penultimate	5.2	43
57	NA	no	265	12	10	ultimate	4.5	29
69	clusterB	yes	406	16	5	no	5.1	112
69	clusterB	yes	406	16	10	penultimate	3.5	103
69	clusterB	yes	406	16	15	ultimate	2.8	111
165	clusterB	yes	756	26	5	no	5.5	37
165	clusterB	yes	756	26	10	no	5.2	42
165	clusterB	yes	756	26	15	no	4.8	43
165	clusterB	yes	756	26	20	penultimate	NA	NA
165	clusterB	yes	756	26	25	ultimate	4.3	47
170	NA	no	364	24	5	no	NA	NA
170	NA	no	364	24	10	no	4.3	42
170	NA	no	364	24	15	penultimate	5.5	35
170	NA	no	364	24	20	ultimate	5.6	35
246	none	no	471	34	5	no	5.4	86
246	none	no	471	34	10	no	5.4	63
246	none	no	471	34	15	no	5.6	68
246	none	no	471	34	20	no	5.7	61
246	none	no	471	34	25	penultimate	5.5	68
246	none	no	471	34	30	ultimate	5.5	68

Note. AxisII: principal diagnosis on Axis II; treatment_2y: treatment in the last 2 years; n _sessions: number of treatment sessions; duration: treatment duration in days; session: session number; end: variable tagging the last two process measurements; HAQ: Helping Alliance Questionnaire (mean score from the patient's version); OQ: Outcome Questionnaire-45 (total score). NA: missing value.

Supplementary Table 2. Irregular longitudinal data arranged in wide format (same data as in Table 1)

ID	...	n_sessions	OQ_pre	OQ_post	HAQ_1	OQ_1	HAQ_2	OQ_2	HAQ_3	OQ_3	HAQ_4	OQ_4	HAQ_5	OQ_5	HAQ_6	OQ_6
57	...	12	86	9	5.2	43	4.5	29	<NA>	<NA>	<NA>	<NA>	<NA>	<NA>	<NA>	<NA>
69	...	16	127	120	5.1	112	3.5	103	2.8	111	<NA>	<NA>	<NA>	<NA>	<NA>	<NA>
165	...	26	47	37	5.5	37	5.2	42	4.8	43	NA	NA	4.3	47	<NA>	<NA>
170	...	24	61	21	NA	NA	4.3	42	5.5	35	5.6	35	<NA>	<NA>	<NA>	<NA>
246	...	34	100	NA	5.4	86	5.4	63	5.6	68	5.7	61	5.5	68	5.5	68

Note. n_sessions: number of sessions; OQ_pre, OQ_post: OQ-45 total score at the beginning and at the end of the treatment; HAQ_1, OQ_1 . . . HAQ_6, OQ_6: HAQ and OQ-45 scores from the first (5th session) to the sixth process measurement (30th session); NA: regular missing value; <NA>: non-existing missing value.

Supplementary Table 3. Irregular longitudinal data summarized in wide format (same data as in Tables 1 and 2)

ID	...	n_sessions	OQ_pre	OQ_post	HAQ_pu	OQ_pu	HAQ_u	OQ_u	OQ_incpt	OQ_slope	HAQ_incpt	HAQ_slope
57	...	12	86	9	5.2	43	4.5	29	57.00	−2.80	5.90	−0.14
69	...	16	127	120	3.5	103	2.8	111	109.67	−0.10	6.10	−0.23
165	...	26	47	37	NA	NA	4.3	47	35.89	0.46	5.78	−0.06
170	...	24	61	21	5.6	35	5.9	23	59.18	−1.44	3.98	0.08
246	...	34	100	NA	5.5	68	5.5	68	77.20	−0.47	5.43	0.01

Note. n_sessions: number of sessions; OQ_pre, OQ_post: OQ-45 total score at the beginning and at end of the treatment; _pu; score at the penultimate process measurement, _u: score at the ultimate process measurement; _incpt, _slope: individual intercept and slope. NA: missing value.
